# Diagnosis and treatment of post-acute myocardial infarction ventricular aneurysm: A review

**DOI:** 10.1097/MD.0000000000043696

**Published:** 2025-08-08

**Authors:** Hongli Ma, Haiyan Xu

**Affiliations:** a Department of Cardiology, Fuwai Hospital, National Center for Cardiovascular Disease, Chinese Academy of Medical Sciences and Peking Union Medical College, Beijing, China.

**Keywords:** acute myocardial infarction, ventricular aneurysm, ventricular pseudoaneurysm

## Abstract

Ventricular aneurysm (VA) is a severe mechanical complication commonly associated with acute myocardial infarction. Patients who develop VA face a heightened risk of heart failure, arrhythmias, thromboembolism, and heart rupture, resulting in a poor prognosis. The fundamental distinction between VA and ventricular pseudoaneurysm (VPA) lies in whether the heart has ruptured. Medical treatment of symptomatic VA is limited, and surgery repair is frequently the preferred approach. Transcatheter closure techniques serves as a minimally invasive alternative, particularly for patients who are poorly tolerant to surgical procedures. Multi-modality imaging examinations are crucial for the diagnosis and treatment. The optimal treatment plan is formulated after assessment by a specialized cardiac team. Ventricular aneurysm represents a superior prognosis compared with VPA. The management of VA should differ significantly with VPA. At present, there remains a dearth of clinical research on VAs, where substantial clinical randomized controlled trials are lacking. It is therefore imperative that additional clinical studies be conducted in order to provide evidence-based medical guidance for the diagnosis, treatment, and prognosis of VA and VPA in the future. This article aims to provide a thorough review of VA’s pathogenesis, diagnostic techniques, treatment options, and prognosis, offering valuable insights for clinical practice and future research.

**Key ponits**

- The key difference between VA and VPA is whether the heart has ruptured.
- Multi-modality imaging examinations are crucial for the diagnosis and treatment of VA.
- LVAD implantation, interventional therapy, and other options provide more treatment choices for patients who cannot tolerate traditional surgery.

## 1. Introduction

Mechanical complications of acute myocardial infarction (AMI) involve ventricular septal perforation, ventricular aneurysm (VA) formation, rupture of the ventricular free wall, and rupture or dysfunction of papillary muscles. VA often occurs in patients with coronary heart disease following a large area of AMI. It is characterized by outward bulging of a portion of the myocardium, exhibiting a banded, sacculated, or irregular pathological change, along with absence or abnormal movement of the ventricular wall. Previous studies have reported the incidence of VA in AMI patients to be 5% to 15%,^[1]^ predominantly affecting the left ventricle. However, due to the widespread use of percutaneous coronary intervention (PCI), the occurrence of left ventricular aneurysm (LVA) following ST-segment elevation myocardial infarction (STEMI) has significantly decreased. The American Heart Association/American College of Cardiology (AHA/ACC) predicts that the incidence rate of LVA is now below 5%.^[2]^

## 2. Methods

This narrative review was conducted by searching the PubMed, Google Scholar, and SinoMed databases using the terms “ventricular aneurysm” OR “ventricular pseudoaneurysm.” Relevant literature on pathophysiology, imaging diagnosis, treatment options, and clinical outcomes was reviewed and synthesized. As this review is based solely on previously published studies, no ethical approval or patient consent was necessary.

### 2.1. Etiology and classification

VA is classified into congenital or acquired based on their etiology. Acquired VA is mainly caused by cardiomyopathies, myocarditis, parasitic infections, trauma, iatrogenic diseases, cardiac sarcoidosis, and AMI. Among these, AMI is the most common cause. Distinction between VA and ventricular pseudoaneurysm (VPA) is often necessary in clinical settings, with the primary distinction being the presence of a heart rupture (Table 1).

**Table 1:** Distinguish between ventricular aneurysm and ventricular pseudoaneurysm.

	Ventricular aneurysm	Ventricular pseudoaneurysm
Pathogenesis	Myocardial infarction, congenital disease, cardiomyopathy, infection.	Myocardial infarction, iatrogenic injury, trauma.
Pathophysiology	Infarcted myocardium bulges outward.	Ventricular rupture confined to the pericardium.
Location	Anterior and parietal walls.	Lateral and posterior walls.
*Gender*		
Female	++	+
Male	+	++
Post-myocardial infarction incidence	5–15%	0.2–0.3%
Onset time	Within 3 months	Within 5 days
Hemodynamic impact	+	+++
*Imaging evaluation*		
Aneurysmal neck	Wide	Narrow
Ventricular echo	Continuation	interruption
Intramural thrombus	+	+++
Treatment	Medicine, selective operation	Emergency operation
Mortality	+	+++

+: mildly present; ++: moderately present; +++: highly present.

### 2.2. VA

Patients with true aneurysms commonly present with angina pectoris, heart failure (HF), and arrhythmias.^[3]^ VAs lose their systolic function due to transmural necrosis and are connected to the rest of the ventricular wall by a wide neck. VA is characterized by a protruding ventricle that does not contract, consisting of fibrous tissue, necrotic muscle, and occasionally a combination of surviving heart muscle.^[4]^ The formation of VAs may be attributed to changes in the geometric and structural arrangement of muscle cell bundles, as well as the enlargement and thinning of necrotic areas caused by cell slippage and stretching, resulting in increased cardiomyocyte loss and fibrosis. VAs can be classified as functional or anatomical. Anatomical VAs lack trabecular muscles, are clearly separated from normal myocardium, and exhibit local bulges during systole and diastole. On the other hand, functional VA has poorly defined borders with the surrounding normal myocardium, with visible sarcomere structures within the cavity and bulging only occurring during systole. Around 80% of left VAs occur in the anterior and parietal walls, often associated with occlusion of the left anterior descending branch. Approximately 10% to 15% of VAs occur in the lower posterior or lateral wall, secondary to occlusion of the right coronary artery.^[5]^ The apex has a higher incidence of VAs due to the presence of only 3 layers of muscles.^[6]^

Multiple studies have investigated the risk factors associated with the development of VA after AMI. Research has demonstrated that individuals who smoke and experience an MI are at a 1.3 times higher risk of developing VA.^[7]^ Smoking contributes to the development of MI and VA by impairing endothelial function, promoting oxidative stress, and inciting inflammation. Furthermore, heavy smoking not only fosters platelet adhesion and aggregation but also prompts the release of vasoconstrictive substances, leading to coronary artery spasms. In a study involving 16,334 individuals hospitalized for AMI with LVA,^[8]^ it was observed that women exhibited a higher incidence and mortality rate compared to men. Additionally, women received fewer percutaneous PCI, coronary artery bypass grafting (CABG), and surgical repairs for LVA. This discrepancy may be attributed to atypical manifestations of MI, delayed diagnosis and reperfusion, lower frequency of revascularization, and the absence of collateral circulation. Recent study identified female sex, elevated levels of NT-pro BNP, time interval from pain onset to balloon dilation, the presence of initial QS waves on ECG, and the existence of regional wall motion abnormalities in the anterior wall and apex of the left ventricle as independent predictors of early-onset LVA in patients with STEMI.^[9]^ These findings provide crucial insights for the clinical prediction and intervention of LVA following AMI.

### 2.3. VPA

VPA is a rare yet severe complication of MI, which represents a type of ventricular wall rupture. The incidence rate of left ventricular pseudoaneurysm (LVPA) following MI ranges from 0.2% to 0.3%.^[10]^ Additionally, it can occur after surgical procedures, trauma, infection, or invasive medical procedures. The pathogenesis may involve adhesion between the epicardium and pericardium after cardiac rupture, which restricts the movement of the ventricular wall. AMI can lead to the tearing of the ventricular wall, resulting in the formation of a narrow channel at the neck. Recent studies have indicated that the most frequent sites of rupture in VPA are located in the lateral and posterior walls of the left ventricle.^[11]^ Due to the relatively small space in the lateral and posterior walls, as well as the supine position commonly assumed by patients with MI, posterior pericardial adhesions are more likely to occur. VPA is surrounded by pericardium and fibrous tissue, lacking myocardial cells. Elderly individuals, males, hypertensive patients, and those with inferior AMI have a higher risk of developing LVPA.^[12]^

### 2.4. Diagnosis and evaluation

#### 2.4.1. ECG manifestations

VA often display varying degrees of ST-segment elevation on electrocardiography. This elevation may be attributed to mechanical stress resulting from the traction of nearby normal myocardial tissue.^[13]^ Previous studies have indicated that the absence of ST-segment elevation in patients suspected or confirmed to have coronary artery disease is unlikely to be associated with VA.^[14]^ Therefore, the presence of ST-segment elevation (with or without associated Q waves in the same lead) serves as a valuable screening sign. Klein et al proposed 2 rules to differentiate AMI from LVA^[15]^:

(a) If the sum of T-wave amplitudes in leads V1 to V4 divided by the QRS amplitude in leads V1 to V4 is >0.22, this indicates a prediction of acute STEMI.
(b) An amplitude ratio of T-wave to QRS greater than or equal to 0.36 in any lead (V1–V4) is indicative of acute STEMI.

Currently, in clinical practice, the formation of VA is often suspected when one of the following criteria is met:

(a) Convex ST-segment elevation ≥1 mV, with ST elevation ≥1 mV persisting for 1 month.
(b) ST-segment elevation ≥2 mV persisting for 15 days.
(c) ST-segment elevation in ≥3 leads accompanied by abnormal Q waves.

#### 2.4.2. Echocardiography

Echocardiography is a valuable diagnostic tool for assessing ventricular wall motion, detecting intracavitary thrombi, and differentiating between VA and VPA. Echocardiography shows thinning and bulging of the ventricular wall, which is most pronounced during systole. The raised wall segments appear thinner and show paradoxical movements. A notable observation is the existence of a broad “aneurysmal neck” situated at the boundary between the endocardium of the aneurysmal wall and the healthy myocardium. The length of this neck often matches or surpasses the maximum diameter of the aneurysmal cavity. Furthermore, echocardiographic assessment can uncover the presence of swirling motion or disturbed flow within the aneurysmal sac. In clinical practice, VAs exhibit impaired diastolic function, which causes an increase in left ventricular end-diastolic volume. Therefore, clinicians need to be attentive to indications of diastolic dysfunction, which can be evaluated using echocardiographic parameters like the E/A ratio.

VPA appears as a cystic, echo-free cavity located between the ventricular wall and the pericardium, with the presence of thrombosis being common. The wall of this cavity is composed of fibrous pericardial tissue, and it is connected to the cardiac cavity through a narrow neck. The key point in differentiating PVA from VA on echocardiography is the width of the aneurysmal neck. PVAs typically have a narrower neck compared to VAs. Recent studies have found that contrast-enhanced echocardiography can clearly demonstrate thrombus within PVA, facilitating rapid diagnosis.^[16]^

#### 2.4.3. Radiographic examination

X-ray chest radiography is capable of detecting large VAs, which typically manifest as an enlarged cardiac shadow with localized bulging of the left cardiac border. Some studies have indicated that transthoracic echocardiography may not be conclusive in determining the precise location of aneurysms when they are found in the Valsalva or high septal regions.^[17]^ In contrast, computed tomography (CT) and magnetic resonance imaging (MRI) not only offer superior spatial resolution, but also enable clearer visualization of cardiac anatomical structures without being affected by imaging planes or acoustic shadowing. CT can observe myocardial motion through images captured during contraction and relaxation, and in conjunction with three-dimensional reconstruction images, it can assist with the diagnosis and assessment of VAs. On CT images, VAs can be identified by features such as ventricular discontinuity, irregular cystocele, and intracavity thrombus. MRI exhibits high accuracy in diagnosing VAs, with imaging changes including localized thinning of the ventricular wall and paradoxical motion of the ventricular wall. Additionally, some studies have shown that serial MRI plays a significant role in understanding the formation process of LVAs and LVPAs.^[18]^ The characteristics of VAs diagnosed by MRI include:

(a) The aneurysm wall exhibits low signal intensity, with a thickness not exceeding 3 mm.
(b) During diastole, the ventricular wall locally bulges out and becomes thinner. In contrast, during systole, there is no movement or any disco ordinated movement of the ventricular wall.
(c) The aneurysm is connected to the ventricular cavity through a large opening.
(d) Gadolinium delayed enhancement reveals continuous enhancement of the aneurysmal wall along with the normal ventricular wall.

Unlike VAs, VPAs frequently coexist with mural thrombi, resulting in akinesis during both systolic and diastolic phases. Additionally, VPAs often exhibit pericardial enhancement on delayed enhancement MRI. Pericardial inflammation and fibrosis are associated with late gadolinium enhancement in the pericardium. Previous studies have identified extracellular volume and left gadolinium enhancement on cardiac MRI during the acute phase of AMI as predictive indicators for early VA formation.^[19]^ Early MR examination can evaluate subtle changes and the extent of ischemic myocardial edema in the initial stages, providing essential information for reperfusion and restoration of the microcirculation. For surgical procedures, assessing the state of ventricular wall motion and the viability of myocardium within the akinetic wall are significant preoperative assessment metrics, crucial for surgical planning. MR imaging demonstrates high specificity and sensitivity in this regard. Left ventricular end-systolic volume and left ventricular ejection fraction (LVEF) are effective prognostic indicators for patients with VAs.

Ventricular angiography is considered the gold standard for diagnosing VA. During coronary angiography, the extension of coronary arteries into the wall of true aneurysms can be observed, but not into pseudoaneurysms.^[20]^ VPA may exhibit delayed release of contrast agent into the non-contractile space and peripheral coronary artery deficiency.

SPECT myocardial perfusion imaging offers accurate diagnostic capabilities for VA and provides a robust assessment of prognosis in patients with VA, demonstrating a significant negative predictive value. The “inverted eight” phenomenon is a specific sign indicative of VA.

Multimodal imaging plays a crucial role in the diagnostic evaluation and intervention of VA and VPA. This comprehensive approach encompasses CT, CMI, echocardiography, and invasive ventricular angiography.

### 2.5. Treatment

**Figure 1.**
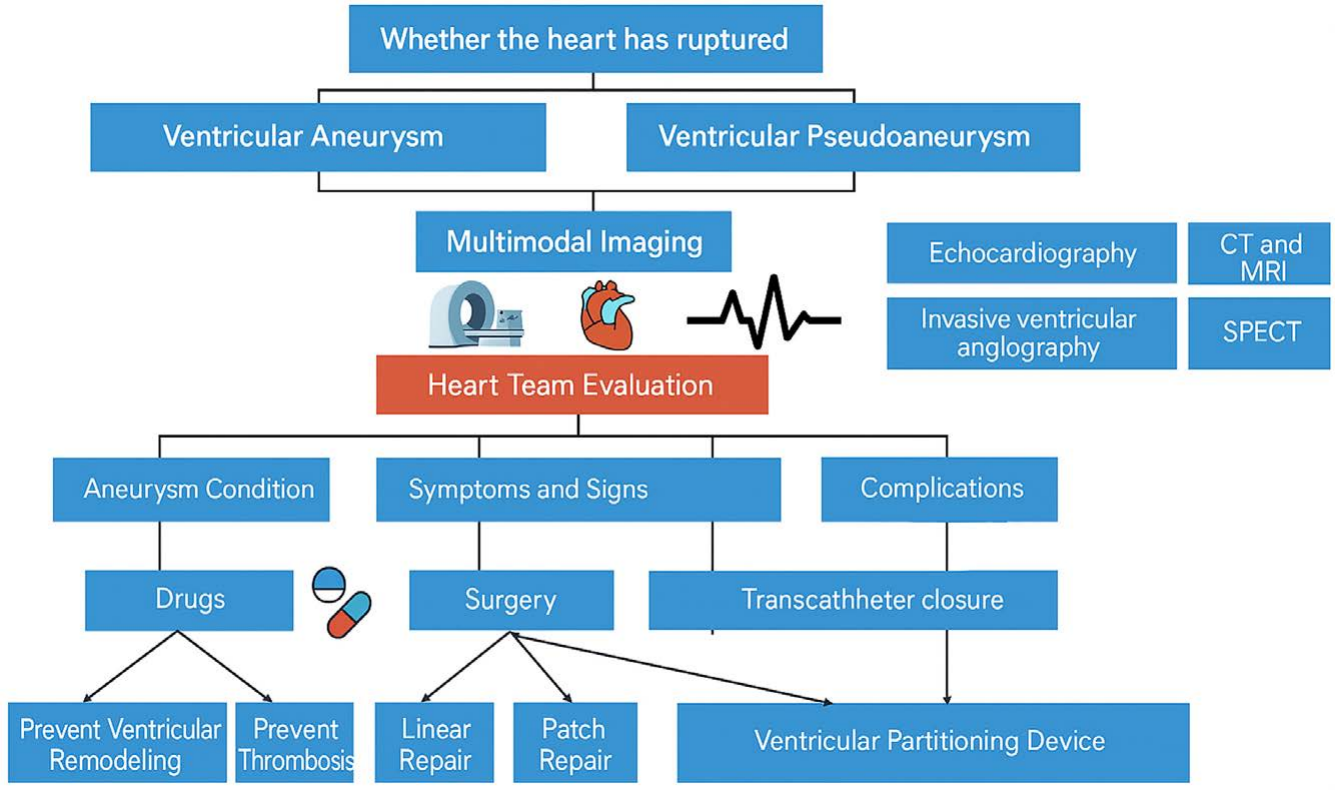
Contemporary diagnosis and treatment scheme of VA and VPA post-MI. The decision process includes multimodal imaging evaluation and heart team assessment, leading to stratified treatment pathways including medication, surgery, or transcatheter intervention.

The treatment scheme for patients with VA or VPA post-MI is illustrated in Figure 1. Management of VA and VPA includes pharmacological therapy, surgical intervention, and interventional therapy. Additionally, ongoing research is being conducted on cardiac rehabilitation, specifically the GRACE study, to examine the impact of cardiac rehabilitation on the prognosis of 100 patients with posterior anterior wall LVA after MI.^[21]^

### 2.6. Medical therapy

In patients with AMI complicated with VA, the contraction dysfunction can reduce cardiac output, further leading to increased left ventricular volume load, mitral regurgitation, and ultimately HF. Pharmacological therapy can help improve cardiac remodeling. For asymptomatic patients with VAs, pharmacological treatment is recommended with regular follow-up exams. The conventional drugs used to treat HF include angiotensin-converting enzyme inhibitors, β-blockers, angiotensin receptor blockers, and aspirin. There are currently no guidelines on whether patients with VA need preventive anticoagulant therapy. For patients with VAs complicated with mural thrombus who have a high risk of recurrence and embolism, anticoagulant therapy can reduce the recurrence of left ventricular thrombosis (LVT), but attention should be paid to the risk of bleeding. The 2013 AHA/ACC STEMI guidelines recommend dual antiplatelet therapy (DAPT) and oral anticoagulant therapy for 3 months in patients with STEMI and asymptomatic left ventricular thrombus, with a target INR range of 2.0 to 2.5 (Class IIa, C). Guidelines from the ACC recommend that for patients with anterior MI at high risk for LVT (LVEF < 40% and abnormal anterior apical wall movement), VKA therapy be performed for 3 to 6 months in addition to DAPT (grade 2C). A retrospective study of 648 LVA patients included 106 patients taking warfarin anticoagulant therapy and 89 patients with LV thrombosis. This study found that warfarin treatment had no significant benefit and was not sufficient to reduce cardiovascular and cerebrovascular events such as systemic embolism. Short-term warfarin prophylactic anticoagulant therapy 3 to 6 months after LVT resection and VA surgery did not reduce the risk of LVT recurrence.^[22]^ Therefore, whether prophylactic anticoagulation therapy should be used still needs further study.^[23]^ For VPAs, for asymptomatic, small (<3 cm), and stable pseudoaneurysm, conservative medical treatment may be attempted under strict detection. Patients without contraindications are routinely treated with DTPA, beta blockers, nitrates, and statins. However, a study analyzed 10 LVPA patients who underwent surgical repair and 7 LVPA patients who only received medical treatment, and concluded that surgical repair is recommended for all patients diagnosed with LVPA, even if it is asymptomatic pseudoaneurysm.^[24]^

### 2.7. Surgical treatment and techniques

Pharmacological therapy has limited impact on the long-term survival of patients with VA, and surgery is recognized as a very active and effective method for treating VAs. Patients with symptomatic VA, such as angina, HF, ventricular arrhythmias, or thromboembolic events, should undergo surgery promptly if they have significant left ventricular dysfunction and reduced ejection fraction. VPA poses a high risk of rupture and may require urgent surgery. On the other hand, VA is mostly treated with elective surgery, aiming to reverse ventricular remodeling and improve HF. The ACC/AHA STEMI treatment guidelines recommend that STEMI patients with VA be considered for LVA resection and CABG (IIa, B) if they have refractory ventricular tachycardia or pump failure that does not respond to drug and catheter therapy. Guidelines recommend surgical treatment for VAs in patients with refractory HF, ventricular arrhythmias uncontrollable by drugs or radiofrequency ablation, and recurrent thromboembolic events despite anticoagulant therapy.

The location and size of VAs vary among patients, leading to a range of repair techniques. In 1958, Cooley et al first performed resection of VA after MI using linear reconstruction techniques assisted by cardiopulmonary bypass. In 1985, Dor and Jatene first reported endoventricular patch plasty (EVPP), which involves the repositioning and suturing of a circular patch within the endocardium. Linear repair techniques and EVPP are currently the main repair techniques for VAs. Linear repair involves resecting the aneurysm and repairing it through horizontal mattress sutures and vertical continuous sutures. However, for larger VAs, there may be excessive reduction in ventricular size. In cases of large VAs or those associated with ventricular septal defects, patch repair is preferred.^[25]^ A study found no significant difference in 1-, 3-, and 5-year survival rates after MI with LVA treated by surgical linear suture or intracardiac patch ventriculoplasty (96%, 91%, 77%, and 96%, 90%, 79%, *P* = .562).^[26]^ The EVPP technique is not always optimal, as a more normal shape can be obtained using simpler linear repair methods for VA with smaller and well-defined fibrotic scars. Surgeons continue to propose and invent new techniques for treating VAs, such as LV volume reduction surgery and preoperative ventricular endocardial repair. For patients with VAs, the goal of surgical treatment is to correct the size and geometry of the ventricle, reduce ventricular wall tension, thereby improving cardiac function and survival rates. The RESTORE study analyzed 1198 patients who underwent surgical ventricular restoration and found a 30-day postoperative mortality rate of 5.3%, significant improvement in LV volume and LVEF, and a 5-year average survival rate of 69%.^[27]^ In patients with coronary artery disease and LVAs who have significant chronic HF symptoms, concurrent LV reconstruction can improve cardiac function and clinical symptoms, significantly reduce the risk of rehospitalization for HF, and improve long-term outcomes compared to coronary artery bypass surgery alone. Recent studies have shown that patients receiving concurrent ventricular septal rupture repair and LVA surgery have no significant difference in long-term outcomes compared to those receiving LVA surgery alone, but patients with VAs complicated by ventricular septal rupture have poor blood perfusion status and related prognosis.^[28]^ In recent years, heart transplantation and cell transplantation have also emerged as potential treatment options for VAs. Given the shortage of heart transplant donors, left ventricular assist device (LVAD) implantation is now a standard treatment for end-stage HF, with the number of LVAD implants outpacing heart transplants annually since 2013. Table 2 presents a chronological summary of studies discussing the surgical treatment of VA and VPA. Research indicates that LVADs are effective for treating VAs, and for patients with advanced HF and VAs, an LVAD with a Dor procedure may be considered to prevent thromboembolic events. There are no guidelines endorsing routine valvular surgery alongside CABG, some studies suggest it could increase postoperative mortality. Traditional LVA surgery relies on cardiopulmonary bypass, which may be too risky for patients with multiple comorbidities, making off-pump techniques a viable alternative.

**Table 2:** Clinical studies in treatment of ventricular aneurysm and ventricular pseudoaneurysm.

Type of study	Author (yr)	Study population	Conclusion
	Reichenspurner H (2020)^[29]^	5	A study involving long-term follow-up of 138 HF patients treated solely with LVAD and an additional 5 patients who underwent Dor surgery concurrently with LVAD implantation suggests that the combined procedure is safe and feasible.
	Ma H (2022)^[21]^	130	Among patients with LVA complicated by MR, there were 77 cases that underwent CABG and LVR, and 53 cases that underwent CAB. There was a significant difference between 2 groups with regard to all-cause mortality (*P* = .019). In terms of MR grading, a substantial proportion of patients in the CABG plus LVR group improved to grades 0 or 1 + (*P* = .030).
	Chen L (2023)^[30]^	12	Circular banding and occlusion technique improved cardiac function in 12 patients with post-MI apical LVA. LVA diameter, LV end-diastolic volume index and end-systolic volume decreased, and LVEF increased.
	Song Y (2023)^[31]^	10	In MI with non-obstructive coronary arteries patients with LVA, EF increased after surgery (*P* = .0009), and no MACE occurred during follow-up.
	Liu Q (2023)^[32]^	254	Among patients with post-MI VA who underwent CABG surgery, survival was significantly reduced in those who also underwent valve surgery (*P* = .00022).
	Clara Großmann (2024)^[33]^	17	In 17 patients who underwent left ventricular reconstruction after Dor, the mean length of stay in ICU was 8 ± 16 days, and the 30-day mortality was 6%. The mean EF was 44 ± 8%.
Prospective, non-randomized observational study	Li J (2021)^[34]^	6	The PARACHUTE device can be used to treat patients with severe left ventricular maladaptive remodeling, but it may increase the HF ratio.
	Briani M (2021) ^[35]^	1	Impella 5.0 was implanted in shock patients with LVA combined with MI as a perioperative LVA support to reduce pulmonary congestion and treat ventricular arrhythmias.
	Stiru O (2021)^[36]^	1	When the mitral valve is unaffected, a trans aneurysmal approach with endoventricular pericardial patch in association with myocardial revascularization is considered a safe option.
	Haidari Z (2022)^[37]^	1	The “high collar technique” is used when LVAD is implanted to provide stable LVAD positioning in patients with LVA, avoiding LV reconstruction and repair plasty.
	Zivkovic I (2022)^[38]^	1	Using extracellular matrix as the material for indoor reconstruction of LVA, instead of traditional polyester, bovine and autologous pericardium.
	Yao S (2024)^[39]^	1	Implanting left ventricular assist device (EVAHEART) in end-stage HF (LVEF 18%) with LVA (61 × 24 mm).

CABG = coronary artery bypass grafting, HF = heart failure, LV = left ventricle, LVA = left ventricular aneurysm, LVAD = left ventricular assist device, LVEF = left ventricular ejection fraction, LVR = left ventricular reconstruction, MACE = major adverse cardiovascular events, MI = myocardial infarction, MR = mitral regurgitation.

For VPA, chronic pseudoaneurysms with small necks can be closed directly by ligation. Studies have revealed a high mortality rate associated with surgery during the acute phase of LVPA. For patients with LVPA who are detected early and have stable conditions, it is considered to postpone the repair surgery to 3 to 4 weeks after AMI under close monitoring of hemodynamics and echocardiography,^[29]^ which aids in the full recovery of infarcted myocardium. However, there is a risk of rupture during the waiting period, requiring evaluation by a specialized cardiac team. Patients with relatively small pseudoaneurysm necks and dense fibrotic margins can undergo direct linear closure. VPA can be repaired using linear, folded and patch prosthoplasty, and the envelope of VPA can be used as a second layer to reinforce the tumor neck. The goal of repair is to preserve the left ventricular geometry while achieving hemostatic repair. The repair of ventricular defects is usually performed in a manner similar to VA repair.

### 2.8. Transcatheter closure techniques

Currently, transcatheter closure techniques primarily involve the use of parachute devices to close VAs. The ventricular partitioning device (VPD), delivered percutaneously through the femoral artery to the left ventricle, can separate the aneurysmal region from the functional ventricle, thereby reducing ventricular volume, decreasing myocardial wall stress, and improving ventricular contractility and mechanical efficiency. Compared to the implantation of cardiac support devices and surgical ventricular remodeling, percutaneous implantation of VPD has the advantage of avoiding the need for revascularization and complications associated with open surgical intervention. In 2014, a study found that VPD implants were beneficial for left ventricular hemodynamics, functional classification, and exercise capacity.^[30]^ A study reported significant improvements in LVEF (32.47 ± 6.98% vs 42.5 ± 7.41%) 1 year after Parachute (a type of VPD) implantation.^[31]^ A study in China was conducted involving 6 patients with LVAD who had undergone PARACHUTE device implantation and had a LVEF ranging from 15% to 40%. After a follow-up period of 4.6 years ± 1.7 years, 50% of the patients experienced major adverse cardiovascular events, while the overall survival rate was 86.7%. However, there was an increased ratio of HF observed.^[32]^

Historically, the treatment of LVPA has been primarily surgical, but many patients are unable to tolerate surgical procedures due to advanced age, frailty, or poor cardiac function. Percutaneous catheter-based left ventricular reconstruction has emerged as a safer alternative. But improper catheter manipulation may lead to device embolism and iatrogenic damage, resulting in severe complications such as pericardial tamponade and cardiac arrest.^[33]^ Clif et al reported the first successful case of percutaneous closure therapy for LVPA in 2004.^[34]^ The main occluding devices used in LVPA include chamber occluding device, chamber occluding device, spring coil, and vascular plug. Recent studies have innovatively employed ASD plugging device and ablation catheter techniques to reduce surgical risks in patients with LVPA.^[35]^ Currently, multiple hospitals in China are conducting clinical trials of the novel occlusion device system Heartech, providing more options for interventional therapy.

## 3. Prognosis

Despite developments in the management of AMI, post-AMI VA, and VPA are still extremely severe condition. Prompt revascularization techniques such as thrombolysis, PCI, and CABG can minimize myocardial cell damage caused by ischemia. Delayed treatment of AMI patients for more than 12 hours can lead to irreversible myocardial damage, reduced local contractility, and increased risk of VA formation due to ventricular wall bulging.

Patients with VAs have a high incidence of LVT, ranging from 14.5% to 30%, and this is strongly associated with systemic thromboembolism. However, the necessity of prophylactic anticoagulant therapy in these cases remains inconclusive. The incidence of ventricular arrhythmias following the development of VAs is not low.^[36,37]^ This is attributed to the presence of islands of viable myocardium in the adjacent areas of the aneurysm and normal myocardial tissue, which serve as the electrophysiological basis for ventricular arrhythmias under various triggering factors. Studies have shown that the incidence of ventricular arrhythmias in patients with LVA is significantly higher compared to patients with MI alone, and it is closely associated with aneurysm size, and moderate to severe mitral regurgitation.^[38]^

Catheter ablation targeting LVA can successfully eliminate most ventricular tachycardias associated with LVA after MI. It is recommended to have regular ECG and rhythm evaluations for patients with VAs to timely detect VA dilation, mitral regurgitation, mural thrombosis, and malignant ventricular arrhythmias.

A study conducted a long-term follow-up on 92 patients with LVA who underwent 99mTc-MIBI gated SPECT myocardial perfusion imaging.^[39]^ It was observed that left ventricular mechanical dyssynchrony is associated with an increased risk of cardiovascular mortality in patients with LVA, with surgical treatment potentially reducing the cardiac mortality rate in these patients (2.40% vs 6.40%, *P* < .05). Additionally, LVEF was identified as an independent predictor of cardiovascular death. In summary, monitoring LVEF and early detection of possible left ventricular mechanical dyssynchrony through GSPECT in the management of LVA patients can guide clinical decision-making.

The risk of pseudoaneurysm rupture decreases over time, with the highest risk occurring on the first day. On the other hand, VAs gradually undergo fibrosis, calcification, and adhesion to the pericardium, making myocardial rupture less common. Untreated VPA rupture rates can reach as high as 30% to 45%.^[40]^ The surgical risk associated with VPA is significant, and it can lead to life-threatening conditions such as pericardial tamponade.

## 4. Conclusion and perspective

VA is strongly correlated with a negative prognosis following AMI. It is imperative to promptly diagnose and treat VA upon the emergence of potential symptoms and signs. First, the study found that women have higher rates of VA morbidity and mortality, while men have higher rates of LVPA, and the reasons for the gender differences in VA and VPA incidence still need investigation. Secondly, the guidelines for patients with LVT recommend DAPT combined with oral anticoagulant therapy, but there is no uniform conclusion on whether VA patients need anticoagulant therapy, and the benefit of anticoagulant therapy and bleeding risk should be balanced. Finally, it is difficult to conduct randomized controlled clinical trials, that require large sample sizes and long-term follow-up. At present, clinical studies mainly focus on case reports and retrospective studies, with low level of evidence and limited guiding significance for treatment. It is therefore imperative that additional clinical studies be conducted in order to provide evidence-based medical guidance for the diagnosis, treatment, and prognosis of VA in the future.

## Acknowledgments

This study was made possible by the financial support provided by Xicheng District Financial Science and Technology Project. Their backing was instrumental in the successful completion of our research.

## Author contributions

**Investigation:** Hongli Ma, Haiyan Xu.

**Methodology:** Haiyan Xu.

**Writing – original draft:** Hongli Ma.

**Writing – review & editing:** Hongli Ma, Haiyan Xu.
